# Topography of the Chimpanzee Corpus Callosum

**DOI:** 10.1371/journal.pone.0031941

**Published:** 2012-02-15

**Authors:** Kimberley A. Phillips, William D. Hopkins

**Affiliations:** 1 Department of Psychology, Trinity University, San Antonio, Texas, United States of America; 2 Southwest National Primate Research Center, Texas Biomedical Research Institute, San Antonio, Texas, United States of America; 3 Department of Psychology, Agnes Scott College, Decatur, Georgia, United States of America; 4 Division of Developmental and Cognitive Neuroscience, Yerkes National Primate Research Center, Atlanta, Georgia, United States of America; University of Edinburgh, United Kingdom

## Abstract

The corpus callosum (CC) is the largest commissural white matter tract in mammalian brains, connecting homotopic and heterotopic regions of the cerebral cortex. Knowledge of the distribution of callosal fibers projecting into specific cortical regions has important implications for understanding the evolution of lateralized structures and functions of the cerebral cortex. No comparisons of CC topography in humans and great apes have yet been conducted. We investigated the topography of the CC in 21 chimpanzees using high-resolution magnetic resonance imaging (MRI) and diffusion tensor imaging (DTI). Tractography was conducted based on fiber assignment by continuous tracking (FACT) algorithm. We expected chimpanzees to display topographical organization similar to humans, especially concerning projections into the frontal cortical regions. Similar to recent studies in humans, tractography identified five clusters of CC fibers projecting into defined cortical regions: prefrontal; premotor and supplementary motor; motor; sensory; parietal, temporal and occipital. Significant differences in fractional anisotropy (FA) were found in callosal regions, with highest FA values in regions projecting to higher-association areas of posterior cortical (including parietal, temporal and occipital cortices) and prefrontal cortical regions (*p*<0.001). The lowest FA values were seen in regions projecting into motor and sensory cortical areas. Our results indicate chimpanzees display similar topography of the CC as humans, in terms of distribution of callosal projections and microstructure of fibers as determined by anisotropy measures.

## Introduction

The corpus callosum (CC) is the largest commissural white matter tract in placental mammalian brains, connecting both homotopic and heterotopic regions of the cerebral cortex [1]. More than 200 million fibers connect the two cerebral hemispheres. Analysis of human post-mortem brain tissue reveals small diameter (<2 µm), lightly myelinated fibers are found mainly in the foremost anterior and posterior regions connecting higher-association areas, whereas large diameter fibers (>2 µm) are predominant in the middle regions connecting primarily motor and somatosensory areas [2], [3]. Furthermore, increased density of axons is found toward the posterior middle and posterior portion of the CC [4].

Knowledge of the origination and distribution of callosal fibers projecting into specific cortical regions is important for several reasons. The CC may be critical to the development and evolution of lateralized structures and functions of the cerebral cortex [5], [6], [7], [8], [9]. In comparative studies of the CC in relation to brain size in mammals, it has been shown that as brain size increases the CC does not keep pace, such that mammals more closely related to humans have a relatively smaller CC after adjusting for brain size than species that are more distantly related [10]. Similar associations have been reported within the primate Order. After adjusting for brain size differences, the primates most closely related to humans, the great apes, have a relatively smaller CC compared to more distantly related primate taxa including Old and New World monkeys [11].It is important to further understand how the organization of the CC in humans differs (or is similar) from other primates. Such knowledge can also be useful for clinical and cognitive studies of CC function, as structural integrity of specific callosal regions is associated with bimanual movements and interhemispheric transfer [12] and neuropsychological performance [13].

Partitioning of the CC has long been geometrically based and not necessarily based upon underlying axonal distribution [14], [15]. Recently, diffusion tensor imaging (DTI) and functional magnetic resonance imaging (fMRI) have been used to identify topographical organization of the CC [16], [17], [18], [19]. DTI provides for the *in vivo* study of fiber tractography through measurement of the displacement of water molecules [20]. In white matter, water displacement is anisotropic, with water diffusion faster along white matter fibers that are parallel rather than perpendicular to these fibers [21], [22], [23], [24]. Diffusion anisotropy measures the difference between these two directions of water motion. One of the most commonly reported measures of this is fractional anisotropy (FA), the normalized standard deviation of the diffusivities [24]. FA values range from 0 to a theoretical maximum of 1; white matter FA values are high, indicating fast diffusivity along the fibers. Diffusion tensor tractography uses the principal diffusion direction to compute the pathways of white matter tracts within regions of interest [25].

No comparisons of CC topography in humans and great apes have been conducted. Here, we used high-resolution structural MRI and DTI to study the topography of the chimpanzee CC. We expected chimpanzees to display topographical organization similar to humans, especially concerning the proportion of callosal projections into the frontal cortical regions.

## Methods

### Subjects

Twenty-one adult chimpanzees (*Pan troglodytes*, 14 male, 7 female), all captive born and housed at the Yerkes National Primate Research Center (YNPRC), were used in this study. The subjects ranged in age from 10 to 39 years (Mean = 20.95 years, SD = 6.68 years).

### Ethics statement

We confirm that steps were taken to ameliorate suffering in accordance with the 93 recommendations of the Weatherall Report. This study was carried out in strict accordance with the recommendations in the Guide for the Care and Use of Laboratory Animals of the National Institutes of Health and were approved by the Institutional Animal Care and Use Committee of Emory University (approval YER-2000090-080313). In order to obtain the noninvasive MRI and DTI images required for this study, the subject's head needed to be immobile during the scan. Therefore, the chimpanzees were anesthetized for the procedure and the collection of MRI and DTI scans was coordinated with the annual physical exam given to the subjects. Anesthesia was used only for the purpose of restraint and to keep the subject immobilized during their physical exam and collection of the brain images. Subjects remained anaesthetized throughout the MRI procedure and respiration rate, heart rate, and oxygen consumption were continually monitored by a veterinarian.

### Image Acquisition

Subjects were initially immobilized using ketamine (10 mg/kg), and subsequently anesthetized with propofol (40–60 mg (kg/h)) following standard procedures at the YNPRC. Subjects were then transported to the MRI facility. Subjects remained anesthetized for the duration of the scan as well as transport time to and from the imaging facility.

Subjects were scanned on a Siemens 3.0 T Trio at the YNPRC. T1-weighted images were acquired using a 3D gradient echo sequence (pulse repetition = 2300 ms, echo time = 4.4 ms, number of signals averaged = 3, matrix size = 320×320, with .6 mm isotropic resolution). The DTI images were acquired following the general procedure employed with chimpanzees previously described by Li et al. [26]. Diffusion-weighted data were acquired with a multishot (4 segments) echo planar sequence with a b value of 1000 s/mm^2^ with 60 diffusion directions. DTI data were acquired transaxially (FOV = 230×230) using 41 contiguous slices with no gap that covered the entire brain with resolution of 1.9×1.9×1.9 mm. Averages of two sets diffusion-weighted data were collected per subject with phase-encoding directions of opposite polarity (left–right) to correct for susceptibility distortion. Acquisition time for both the MRI and DTI scans was approximately 1 hour. After completing the DTI and MRI procedures the subjects were returned to the YNPRC and temporarily housed in a single cage for 6–12 hours, to allow for the effects of anesthesia to wear off, after which they were returned to their home cage and social group.

### Fiber tractography

Each subject's MRI image was spatially registered to their respective DTI image using 3D voxel registration with a linear transformation using Analyze 10.0 (Mayo Foundation for Medical Education and Research). We then used DTI-based fiber tractography to evaluate the projection of callosal fiber tracts between the cerebral hemispheres. Tractography was conducted using Analyze 10.0 MR Diffusion Tensor Imaging based on fiber assignment by continuous tracking (FACT) algorithm [27] with a fractional anisotropy threshold of 0.15 for initial seeding and stopping and a principal eigenvector angle stopping threshold of 40°. These tracking thresholds are similar to those used in fiber tracking studies of the human CC [28], [29], [30], [31]. An ROI approach was used to determine the fiber topography of the CC. The entire CC was manually traced in the transverse plane.

### Partitioning of the CC and Fractional Anisotropy Measures

The CC was subdivided into regions based upon the results of tractography. The CC was partitioned to identify transcallosal projections into prefrontal, premotor, primary motor, primary sensory, parietal, temporal and occipital cortical areas. Identification of clusters of CC fibers projecting to defined cortical regions was determined following Hofer et al. [32], Petrides and Pandya [33], and Ramnani et al [34]. Landmarks used to define projections into cortical areas were the arcuate sulcus (prefrontal cortex); arcuate sulcus and central sulcus (premotor and supplementary cortex; motor cortex; sensory cortex); postcentral sulcus and parietooccipital sulcus (parietal cortex), lateral fissure (temporal cortex); and inferior occipital sulcus and parietooccipital sulcus (occipital cortex). From this, the CC was subdivided into 5 regions based on fiber projections into specific cortical areas and defined as follows: I = prefrontal lobe, II = premotor and supplementary motor cortices, III = motor cortex, IV = sensory cortex, V = parietal, temporal, and occipital lobes. Fractional anisotropy (FA) was evaluated in the midsagittal and two CC sections 1 mm lateral to the midsagittal as a means of quantifying the measures of diffusion anisotropy. Obtained values were averaged for each subject for each callosal region. To test for regional differences in FA, a within-subjects ANOVA was conducted, with Bonferroni correction for post hoc comparisons with alpha set at 0.05.

## Results

### Topography of the CC

**Figure 1:**
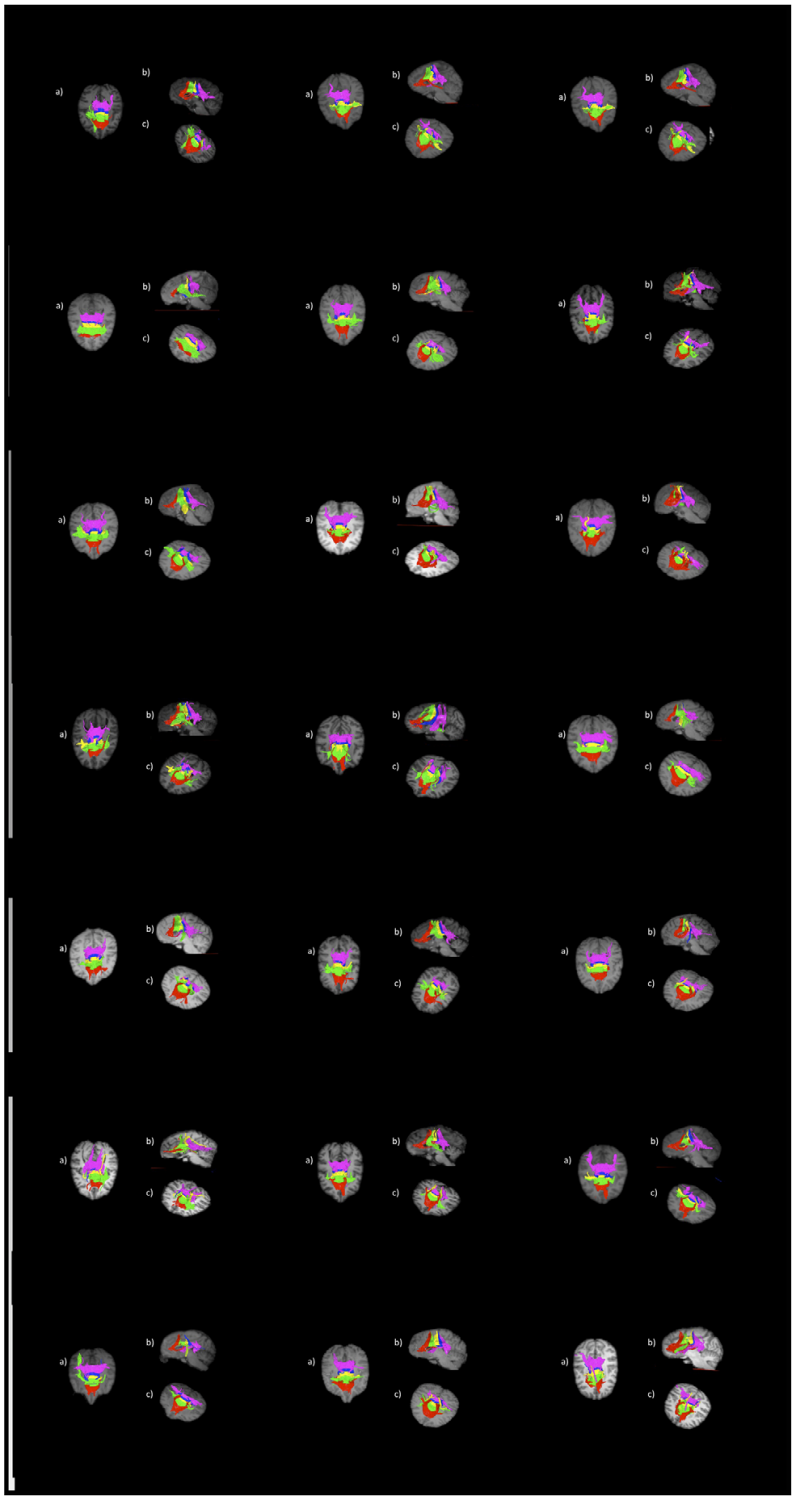
Callosal fiber projections from all 21 chimpanzees, displayed from a) dorsal, b) sagittal and c) oblique views. Color distinguishes fibers projecting into cortical regions and are as follows: prefrontal (red), premotor and supplementary motor (green), motor (yellow), sensory (blue), and parietal, temporal and occipital (violet). For ‘a’ and ‘c’, the right side of the brain is on the left side of the image.

The topography of callosal fiber bundles is displayed in Figures 1 a, b, c. These figures, from all individual chimpanzee subjects, illustrate from sagittal, oblique and dorsal views the projection of callosal fibers into cortical regions. Regions I, II and III encompass all cortical projections into the frontal lobe and accounted for, across all chimpanzees subjects, approximately 64% of the length of the CC.

### Regional differences in FA

**Figure 2:**
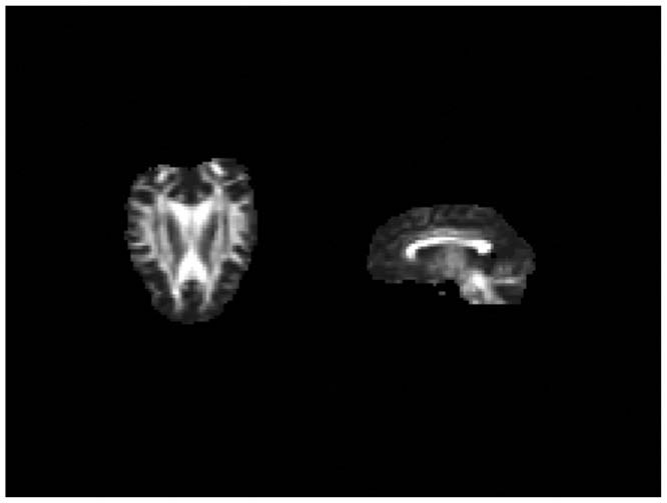
Mean fractional anisotropy map from 21 chimpanzees.

**Figure 3:**
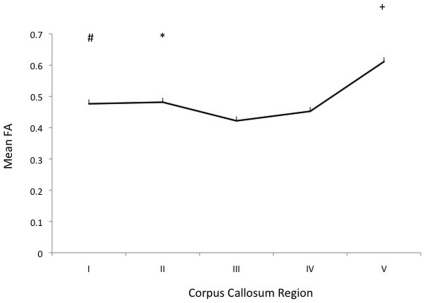
Mean fractional anisotropy measures by corpus callosum region in 21 chimpanzee subjects. Region I = prefrontal; region II = premotor and supplementary motor; region III = motor; region IV = sensory; region V = parietal, temporal and occipital cortices. The pound (#) indicates a significant difference between Region I and Region III (*p* = 0.032); the asterisk (*) indicates a significant difference between Region II and Region III (*p* = 0.028); the plus (+) indicates significant differences between Region V and Regions I (*p*<0.001), II (*p* = 0.013), III (*p*<0.001) and IV(*p*<0.001).

Quantitative measures of diffusion anisotropy were evaluated for each subdivision of the CC. The mean FA map of the 21 chimpanzees used in the study can be seen in Figure 2. The highest FA values were found in the posterior region (region V, M = .61, SE = 0.01), followed by the premotor and supplementary motor region (region II, M = .48, SE = .02) and prefrontal region (region I, M = .48, SE = .01). The lowest FA values were found in the middle regions of the CC, regions III and IV, which project to primary motor and primary sensory cortical regions. FA values were significantly different across partitions of the CC (*F*(4, 80) = 25.93, *p*<0.001; Figure 3). Bonferroni comparisons revealed region I to have significantly higher FA value than region III (*p* = 0.029); region II to have significantly higher FA value than region III (*p* = 0.020); and region V to have significantly higher FA value as compared to all other regions (region I, *p*<0.001; region II, *p* = 0.005; region III, *p*<0.001; region IV, *p*<0.001). There were no significant effects of sex (F (1, 19) = .011, p = .919), or an interaction between sex and callosal subdivision (F (4, 76) = 1.23, p = .304).

## Discussion

Based on this DTI study, chimpanzees display similar topography of the CC as humans and rhesus monkeys, based upon the percentage of callosal projections into the frontal cortices. Chimpanzees were shown to have 64% of callosal projections to frontal lobe. In humans, projections to the frontal lobe account for approximately 67% of callosal projections [17]. Rhesus monkeys, the only other primate to date for which callosal projections have been mapped, have 60% of callosal projections to the frontal lobe [32]. Thus, considerable homology in the cortical projections of fibers running through the CC is seen -among all three species. -

Chimpanzees also showed similar FA patterns to humans in that the lowest FA values were seen in cortical projection areas into motor and sensory regions [17]. One difference between chimpanzees and humans concerns which subdivisions had the highest FA values. In chimpanzees the highest regional FA value in the CC was in region V, followed by I and II. All of these regions are comprised of fibers projecting into the higher-association areas of prefrontal, premotor and supplementary motor cortical areas and posterior cortical areas (including parietal, temporal and occipital cortices). Region V connects areas of the parietal, temporal and occipital lobes known to be involved in the visual processing of complex spatial tasks [35]. As FA is an index that reflects the degree of directionality and coherence of fiber tracts [24], this indicates that the chimpanzee CC is characterized by highest white matter organization in CC regions involved in higher-association areas. These regions likely contain small diameter, lightly myelinated fibers but histological examination of the chimpanzee CC is necessary to confirm (but see [36], [37]). Regional differences in signal intensity obtained via structural MRI scans have been used as a means of quantifying the degree of myelination [38], [39]. Regions with greater myelination are expressed with higher voxel intensities than regions with less myelination, such as grey matter. Within the chimpanzee CC, the most anterior and posterior region, the genu and splenium, had the highest signal intensity values; the lowest signal intensity was reported in the middle portion of the CC [39]. These results reveal a similar pattern to regional FA obtained in the present study, indicating that signal intensity from MRI does potentially provide reliable information concerning myelination.

When comparing the partitioning of the CC based on the results of tractography to the geometric schemes typically used with structural MRI or on post-mortem material, a greater proportion of the CC includes areas that project to the frontal lobe. Using Witelson's scheme, a widely used method for subdividing the CC, the most anterior half of the CC is considered to project to the frontal lobe. The results of our analysis indicate that in the chimpanzee approximately 64% of the fibers of the CC have projections to the frontal lobe. Our method confirms recent studies by Hofer and colleagues [17], [32] that using fiber tractography results in a refinement of the parcellation of the CC and more precisely accounts for projections to the frontal lobe. This observation is by no means trivial because much has been written regarding sex and handedness effects in relation to variation in the size of different CC regions in human and nonhuman primates [40], [41], [42], [43], [44]. Whether these dimorphisms in the CC are related to the number or distribution of axons traversing callosal subdivisions, or differences in myelination of these axons, is unknown. Data concerning the relationship between handedness and anisotropy are inconsistent in humans, likely due to confounds such as age [29], pathology [45], and musical training [46]. Similar inconsistent results regarding the relationships between CC morphology, sex and handedness have been reported for chimpanzees. While Dunham and Hopkins [43] did not detect sex differences in CC morphology in chimpanzees, Phillips et al. [39] reported sex differences in both CC size and signal intensity (in MRI, higher voxel intensity reflects the level of myelinated axons). We did not find sex differences in FA values of callosal subdivisions in the present study. However, this may be due to the unbalanced distribution of males and females (male *n* = 14; female *n* = 7). We were unable to investigate the effects of sex and handedness in the present study as hand preference data on bimanual tasks in these subjects is not complete. However, given the importance of such data to further our understanding of the relationships between CC morphology, sex and handedness, and the role of the CC in hemispheric specialization, such data are needed. Many have employed the CC divisions proposed by Witelson as a foundation for interpreting sex and handedness differences, which have often been inconsistent across studies.

As shown in this study, the topographical organization of the chimpanzee CC is similar to humans. Chimpanzees and humans also display marked similarity in the development of the CC and callosal subdivisions [47], as well as the prefrontal cortex [48]. Comparative analyses of primate neuroanatomy are essential to reveal the neurobiological specializations of humans and apes, and provide insight as to the functional consequences of such specializations.

Collectively, these data indicate that the chimpanzee CC is characterized by highest white matter organization in callosal regions involved in higher-associated areas. Findings, such as those provided here, provide a more empirical functional connectivity framework for dividing the CC into quantifiable anatomical regions that can potentially be correlated with aspects of functional lateralization.
